# Student psychological well-being in higher education: The role of internal team environment, institutional, friends and family support and academic engagement

**DOI:** 10.1371/journal.pone.0297508

**Published:** 2024-01-25

**Authors:** Smita Chaudhry, Ankita Tandon, Shilpa Shinde, Anindita Bhattacharya

**Affiliations:** 1 Department of Human Resources, FLAME University, Pune, Maharashtra, India; 2 OB&HR Area, International Management Institute New Delhi, Delhi, India; 3 Department of Psychiatry and Clinical Psychology, Narayana Health, Bengaluru, Karnataka, India; Politehnica University of Timisoara: Universitatea Politehnica din Timisoara, ROMANIA

## Abstract

Psychological well-being of students is an area of concern in higher education institutes across the world. Although several studies have explored the factors associated with students’ psychological well-being, limited research has focused on the relation between the overall support for students and psychological well-being. Students of higher education may get formal support, in the form of team environment and institutional support; and informal support, in the form of family and friends’ support. The purpose of this study is to examine the relation of these four kinds of support with psychological well-being of management students. We also examine the intervening role of academic engagement in this relationship. Analysis using structural equation modeling and hierarchical regression on data collected from 309 management students from Indian universities, shows that positive internal team environment, and institutional and family support positively relate to students’ psychological well-being. Academic engagement partially mediates the relation between positive internal team environment and psychological well-being, and family support and psychological well-being. Also, academic engagement fully mediates the relation between institutional support and psychological well-being. The study highlights the significance of internal team environment and institutional support for students’ academic engagement and psychological well-being, and the role of academic engagement in determining well-being. Based on these findings, we suggest interventions that can be undertaken by educational institutions to enhance psychological well-being of students. Theoretical implications and research avenues are discussed.

## Introduction

Psychological well-being is a longstanding issue faced by university students worldwide [1–3]. University education is a significant life transition and students need to adjust to several new social, cultural and academic demands. Well-being of students can be affected by several factors including transition to a different pedagogy and tools and techniques for learning, relational support, and education environment. This is especially true for management education, where the course structure is rigorous and intensive, requiring students to attain a breadth of personal and professional skills through in-class and extracurricular activities in limited time [4]. Also, students may be required to be away from home for a long duration. Many of them may also be in residential programs. All these factors can considerably hamper their well-being. Studies on graduate students have revealed that 51% of them experience psychological distress [5], and 40% experience symptoms of mental illness [6]. One of the major factors that can enhance students’ well-being is the formal and informal support they receive from different sources.

Various studies have discussed how social support systems impact student well-being [7–11]. However, there are several gaps in this literature. First, the impact of work environment of assignment teams, a critical pedagogical tool in management education, on well-being is not examined. Second, the role of multiple formal (internal team environment and institution) and informal (family and friends) support systems have not been studied together in a single study. Third, the mediating role of academic engagement in the support-well-being relationship has not been studied. Strong support systems can enable students to take interest, and immerse themselves in academic pursuits that will in turn induce positive emotions, better academic adjustment and higher well-being. Therefore, in this paper, we explore the relation of two sources of formal support (internal team environment and institutional support) and two sources of informal support (family and friends support) with psychological well-being of students of management education, and examine the mediating role of academic engagement in determining these relationships.

Internal team environment (ITE) refers to an environment that facilitates positive experiences in teams, and it comprises of a sense of shared purpose, social support and voice in the team [12]. In this paper, ITE pertains to academic teams in which the students perform activities and assignments, across different courses. Institutional support refers to the resources, opportunities and services provided by the educational institute to the students, which can enable them to achieve their individual goals [13]. Family and friends support encompass the social support available to the students. Social support refers to socioemotional, instrumental and informational aid provided by family members and friends [14]. Academic engagement refers to the student experience of high enthusiasm, energy, perseverance and deep engrossment in academics and related activities [15, 16].

ITE is especially relevant for management education where pedagogy is heavily driven by teamwork [17]. Team assignments simulate the dynamics of organizational teams and provide experiential knowledge of working together for problem solving, collaborative learning [18], and developing peer networks. This accelerated student-driven mode of instruction, although highly engaging, is also very stressful and makes students vulnerable to higher psychological distress and lower well-being [19, 20]. When students spend considerable amount of time working in teams throughout the program, it is likely to have a significant impact on their mental health. Studies indicate that cooperative team environments and positive peer relationships affect mental health positively, as they lead to goal achievement [21]. Conversely, students experience dissatisfaction and negative affect when there is uncooperative behaviour and social loafing in teams. as they impede goal achievement [22]. Most literature on ITE, to our knowledge, is in the context of shared leadership [12, 23]. However, there is limited literature on the impact of ITE on the psychological well-being of management students. In this paper, we address this gap.

Existing studies have indicated the positive impact of institutional and family and friends support on well-being [7]. Support provided by the educational institution, through its processes and policies, can impact student well-being by creating a safe and positive environment [8, 24]. Besides, when young adults feel supported by family and close friends, they develop a sense of belonging at the academic institution [25], cope with their lives better [26], and adapt more to the educational environment [27]. Incidences of mental illness amongst students are also reduced [28]. This paper explores the relation of institutional, family and friends support, and support provided by ITE, with psychological well-being of management students. We do not know of any literature which has examined these formal and informal support systems in the context of well-being from a holistic perspective.

Positive experiences of working in teams is likely to result in positive emotions like higher involvement, interest, and immersion [12]. Similarly, institutional, family and friends support also create positive emotions. These positive emotions can enhance engagement levels by building personal resources [29, 30], and subsequently well-being according to broaden-and-build theory [1, 31]. Thus, existing studies suggest an intervening role of academic engagement in enhancing well-being in the context of support systems. However, to our knowledge, the influence of academic engagement on well-being is not clearly established in literature [32, 33]. We address this gap by examining the mediating role of academic engagement in the relationship between formal and informal support systems and psychological well-being.

Thus, this paper explores the relation of ITE, institution, family and friends support with students’ well-being, and examines the mediating role of academic engagement. We chose to collect data from management students as management education can be very stressful due to its intensive and rigorous curriculum, concurrent student-driven extracurricular activities, and the pressure of performing well in both within a limited time frame. The pedagogy is heavily driven by team assignments thus making it relevant to examine the role of ITE. For this study, data was gathered from 309 management students in Indian universities using an online questionnaire survey. Since our objective was to examine the causal relationships between the variables, we employed structural equation modeling to analyze the data. The study contributes to literature by developing a comprehensive understanding of the various support systems (formal and informal) that impact students’ psychological well-being. It brings forth the role of ITE, a critical variable whose impact on well-being has not been examined. It highlights the importance of institutional support in student well-being, which has been given limited attention in literature. The results show that all sources of social support are not equally effective in impacting wellbeing. While family support matters, friend support may not always influence wellbeing positively. The study furthers literature by showing that academic engagement is not only relevant for performance but also for psychological well-being of students. Formal and informal support systems can help students experience higher well-being through enhanced academic engagement. We suggest that schools and teachers develop academic practices to promote positive team environment in assignment teams, and institute level support systems which signal that the institute cares for students’ wellbeing. Lastly, our inferences are limited by the cross-sectional nature of our data and can be further strengthened through a longitudinal study. We discuss all these aspects in detail in the sections that follow.

## Theory and hypotheses

### Psychological well being

Well-being is a multidimensional construct consisting of psychological, emotional and social aspects. Psychological well-being (PWB) comprises six dimensions: a sense of purpose in life, striving towards personal growth, a sense of confidence in influencing the environment (environmental mastery), acceptance of oneself for who they are (self-acceptance), independence and self-agency regarding one’s thoughts and actions (autonomy) and fruitful relationships with others (positive interpersonal relationships) [34]. These well-being domains reflect a great degree of access to coping resources that are useful in dealing with psychological stress. PWB is essential in promoting intrinsic objectives, meeting fundamental emotional needs, being aware and doing with consciousness, and performing independently [1, 35].

In positive psychology, there is a focus on well-being and personal strengths aligning itself with aims of education as stated by [36]: learning to know, learning to do, learning to be and learning to live together. Personal well-being scores early in college life positively predicted students’ personal growth and academic achievement after three years [37]. Students with positive well-being are likely to perform better [38], and achieve academic success in terms of grades, credit accumulation and persistence [25], while negative well-being factors such as mental illness and mental distress, are likely to impact performance and course completion negatively [39].

Several factors have been associated with students’ PWB like anxiety [40], hope [41], perceived social support [42], student-supervisor relationship [43] and authentic assessment [44].

**Fig 1:**
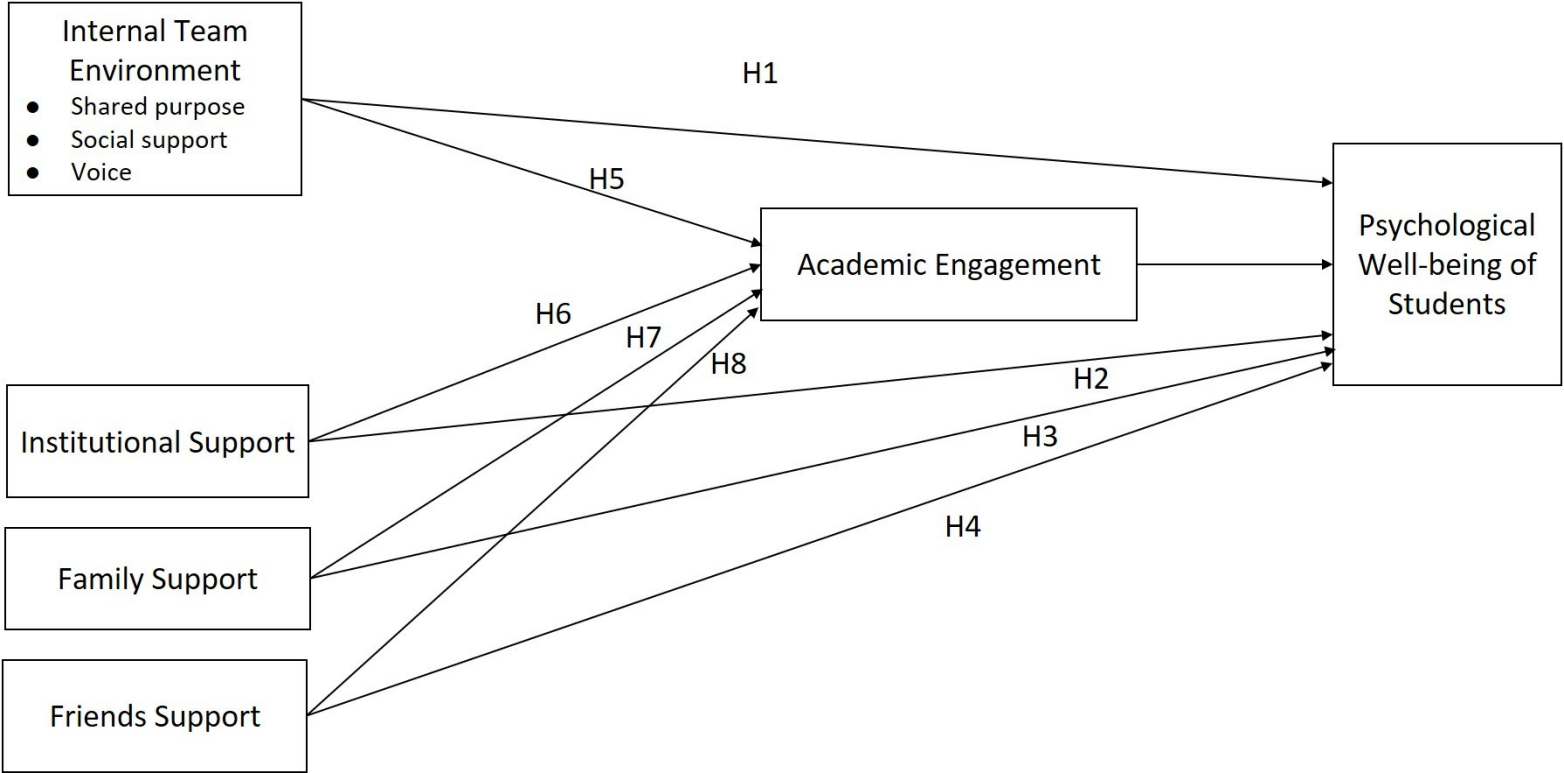
Conceptual model of the factors determining students’ psychological well-being.

The following paragraphs present arguments on how internal team environment, institutional support, family support and friends support may impact psychological well-being of students directly, as well as through academic engagement. Fig 1 depicts the conceptualized model.

### Internal team environment

ITE refers to conditions within a team that enable team members to have a favorable experience while working on a task [12]. ITE includes three components: shared purpose, social support and voice. Shared purpose refers to the commonality of the purpose and goals across team members. Social support refers to the informal mutual support received by team members while performing a team activity. Voice refers to the comfort and ability of the team members to be vocal about their thoughts, ideas, preferences and concerns. A positive ITE promotes shared leadership, cohesion, and constructive conflict [12, 23, 45]. It enhances knowledge sharing [46] by promoting a culture that aligns individual behavior towards team goals [47]. In contrast, a negative inter-personal environment can have harmful effects like knowledge-hiding [48].

The role of student teams in management education is well recognized. Studies in the domain have examined the role of collaborative learning, the tools used, the process of teamwork, curriculum design and alignment with learning outcomes, team composition factors, and issues related to assessment [49]. The outcomes of working in student teams include positive academic outcomes, engagement and soft skill development. However, the role of these teams in impacting well-being is largely overlooked. Collaborative learning in teams enhances communication skills, leadership skills, problem solving [50], teamwork and critical thinking skills [51], and promotes academic engagement [52]. At the same time, there is sufficient contradictory evidence regarding the perceived benefits of teamwork due to group processes such as social loafing and inability to manage group conflict [17, 49]. Students may experience dissatisfaction with work teams if the teams are high on social loafing and the team process does not enable them to meet their academic goals [22]. Given that management students spend extensive amounts of time working in groups, their experiences in the team and its outcomes can have a significant impact on their mental health. This can lead to negative affect which can impact well-being negatively. Alternatively, experiencing group processes which support individual and group goal achievement can enhance team satisfaction, increase positive affect and promote well-being. Effective teams leading to positive outcomes require group processes focused on working towards shared goals, mutual support and accountability [17]. This is captured in the concept of ITE.

According to the social interdependence theory [21, 53], individual goal achievement is dependent on the actions of others. Interdependence can be positive (i.e., when the individual’s goal attainment is positively linked with the attainment of goals by others), negative (i.e., when the individual’s goal attainment is linked with failure to achieve goals by others), or there can be no relationship between the goal attainment of the individual and the others [53]. Students in assignment teams are linked together by positive interdependence. Positive interdependence promotes cooperation and collaboration. Literature has found an association between attitude and well-being [54]. Cooperative attitudes in teams enable positive relationships with peers and positive psychological health including higher self-esteem, trust and optimism, and positively adjusted social relationships [21]. Learning in collaborative setups has a positive impact on well-being [50]. Additionally, the self-determination theory posits that work environments which fulfill an individual’s competence, relatedness and autonomy needs have a positive impact on well-being [55]. A positive internal team environment can fulfill these needs leading to higher well-being.

A positive ITE enables psychological, social and emotional support in student teams [23]. A shared purpose within the team provides meaning and direction to the team activities. This can make students convinced about investing time and effort in the team. When students realize that their team members care about their well-being and value their contribution, they are likely to invest greater efforts towards team goals [56] thus increasing cooperation, possibilities of learning from others, and getting help when required. It can also help in sharing each other’s thoughts and feelings, which can make them more trusting of each other, and lead to positive interpersonal relationships. Use of voice within the team can help the students openly share ideas, suggestions and perspectives. It can make them feel psychologically safe about raising issues and sharing doubts [57]. Being vocal without fear can give them a sense of autonomy and confidence about influencing the environment. A positive team environment improves group cohesion and unity while minimizing interpersonal conflict and detrimental disagreements [23]. All these factors can enhance the psychological well-being of the students. Therefore, we propose the following:

*Hypothesis 1*: *A positive internal team environment is positively related to psychological well-being of students in a team*.

### Institutional support

Institutional support (IS) refers to the support provided by the educational institution to its students [8, 24]. It includes highly valued resources, opportunities and services that senior and powerful agents of the institution provide to its people to promote their development and success and make them effective participants in the institution [13].

IS is conceptually derived from perceived organizational support (POS). POS is based on the organizational support theory which argues that employees tend to anthropomorphize their organizations [58]. They develop perceptions of organizational support based on actions of individuals with whom they interact. They consider these actions as indicators of organizational intentions as they believe that organizations have full responsibility for those actions because of the vested authority to take such actions, and create the policies, norms and culture that aids such actions. POS makes employees feel that the organization values and cares about them. It mitigates negative mood in stressful jobs and enhances PWB [59, 60]. Studies have discussed the impact of elements of POS like leadership and climate on employee behavior [61].

Unlike business organizations, students’ perception of IS is based purely on the support provided by the institution and not by the faculty [8]. IS determines the ease with which they can navigate through day-to-day academic life. It also conveys the care, consideration and commitment the institution has for the students. IS increases student satisfaction, academic performance [24] and psychological well-being [9]. Conversely, poor IS can lead to academic burnout characterized by exhaustion, loss of interest, poor engagement, dampened emotions and a sense of helplessness [62]. Perceptions of psychological support reduces stress and the risk of burnout among students [63]. Perceptions that one matters to the institute can reduce academic stress of the students and enhance their wellbeing [64]. Academic, career and psychological support along with understanding student concerns and a positive environment provided by the institution are linked with higher students’ well-being [65, 66]. Displaying empathy, promoting mental health awareness, giving heed to student voice, and adopting a whole person approach are some of the ways in which institutions can enhance student well-being [67]. Making institutional support proactive, easily accessible, and creating safe spaces for student voice and expression can further improve student well-being [68]. Therefore, we propose the following:

*Hypothesis 2*: *Institutional support is positively related to psychological well-being of students in a team*.

### Family and friends support

Family support (FAS) refers to the emotional and psychological support and care provided by family members [69, 70]. Friends support (FRS) refers to the emotional and psychological support provided by friends who can be classmates and peers within or outside the educational institution. FAS and FRS together constitute social support for students. We distinguish social support based on FAS and FRS from the social support dimension of ITE. Social support in ITE is focused on supporting team members to complete the task at hand. It is task and team goal focused. Further, team members are not always likely to be close friends and may not provide long term socio-emotional support. In contrast, FAS and FRS focuses on long term support which encompasses all aspects of a student’s life experience.

The PERMA model of well-being highlights relationships as a central building block of well-being [71, 72]. Positive relationships make individuals feel that they are cared for, loved and supported by others [71]. Similarly social support theories indicate that integration in a social network and strong relationship ties within it enhance positive affect, sense of self-worth and give a sense of stability [73]. Social support also acts as a buffer by reducing the impact of stressors in stressful situations by providing resources to mitigate them, reducing and eliminating the reactions to stress, providing solutions and reducing the perceived importance of the stressor. Thus, social support including both friends and family is related to higher well-being [10, 11, 74, 75].

Social support provides cues to the individual that she/he is cared for, valued and included in a social network [69, 71]. It creates a supportive environment and acts as a buffer in stressful situations [76], enhances quality of life and impacts mental health positively [7]. In a study with at-risk adolescent girls and boys, it was found that experiencing FRS and FAS reduced subsequent depressive symptoms [77]. Social support reduces the perception and experience of academic stress [27, 78]. It leads to perceptions of opportunities which support educational and career aspirations [79]. It enables personal adjustment [80] and improves academic performance [76] even in adverse and new situations.

FAS is an important factor in academic performance [81] and academic adjustment [82]. Positive relationships and support from family helps with reducing anxieties and self-doubt and enhancing efforts towards building skills, enabling academic performance. In universities, development of friendships supports student integration into the educational setup through developing a sense of belongingness [27].

Overall, these studies indicate that FAS and FRS enhance wellbeing [19, 79]. They have found that the extent of emotional support received from family is positively related to academic outcomes of students and this relationship is explained through the contribution of psychological well-being [25, 83]. Thus, we expect that family and friends support will be positively related to PWB of students working in teams. Therefore, we propose the following:

*Hypothesis 3*: *Family support is positively related to psychological well-being of students in a team*.
*Hypothesis 4*: *Friends support is positively related to psychological well-being of students in a team*.

### The mediating role of academic engagement

Academic engagement has been defined as “a positive, fulfilling, and work-related state of mind that is characterized by vigor, dedication, and absorption” [16]. In this definition “work” refers to the activities performed by students [84]. Therefore, academic engagement happens when students experience high levels of energy, mental resilience, willingness as well as an ability to invest effort, dedication, a sense of significance in their studies, feelings of pride and challenge, complete concentration and a feeling of being carried away by one’s studies such that time passes quickly [84]. AE is desirable, not only for the performance of students, but also for their learning experience.

When students are engaged, they exhibit interest in classes and find them challenging [85], exhibit greater academic progress [86], demonstrate good academic performance [87, 88] as well as achievement. They are actively involved, pay attention to and are committed to academic activities [89]. A very important outcome for any higher education institution is student persistence and degree attainment. Students showing behaviors that reflect academic and social engagement, are found to be more likely to persist and complete their degrees [90].

However, more studies are needed to explore the antecedents of study engagement among university students [87].The influence of psychological well-being and psychological capital on engagement has been studied [91, 92]. There has also been research on the influence of the broader concept of student involvement on psychological well-being [93, 94] as well as the mediating role of engagement on academic performance [95]. However, there are not many studies which throw light on the influence of student engagement on psychological well-being.

As discussed previously, positive ITE is characterized by collaborative learning. Collaborative learning implies peer to peer interactions enabling problem solving, knowledge construction and cooperation towards a common goal [18, 52]. It is found that team based collaborative learning leads to higher involvement in blended courses [96] and enhances peer to peer interactions. A common understanding of shared goals, authentic cooperation and member support can enable student teams towards higher effectiveness [17] through higher involvement in team tasks. Studies have found that collaborative learning impacts academic engagement positively [52]. Besides, according to social interdependence theory, there is a difference between cooperators and competitors. Cooperators show higher levels of academic engagement as compared to competitors, which is indicated by the time they spent on tasks [21]. Thus, we argue that a positive ITE can result in higher engagement through cooperative behaviors. A positive ITE enables greater focus and identification with team goals [97], higher task involvement and immersion [98]. Such a strong identification and involvement with work are essential to the process of flow and engagement. Flow which is an enjoyable state of mind and is characterized by total absorption, is similar to engagement [99]. Studies show that students who experience flow have higher levels of PWB [100]. Thus, positive ITE characterized by cooperative behaviors may lead to PWB through higher engagement levels. Therefore, we propose the following:

*Hypothesis 5: Academic engagement partially mediates the relationship between a positive internal team environment and psychological well-being of students in a team*.

Decades of research on the impact of college on students has found that institutions have a lot to offer in enhancing student engagement [101]. Tinto’s interactionalist theory of individual student departure discusses how various support-oriented academic practices and programs provided by institutions can impact the academic integration and engagement of students [102]. For example, providing students with orientation, learning communities, advising, tutoring, supplemental instruction, peer tutoring, study groups, summer bridge programs, study skills workshops, mentoring and student support groups influence student success [103]. Positive interactions with administrative personnel and services, having information regarding where to get help and access to institutional resources are important sources of institutional support, leading to higher student engagement [104]. Institutional support mechanisms can enhance autonomous behavior and this student autonomy has a positive effect on student engagement [105]. Since academic engagement leads to positive outcomes such as academic performance [15] and academic achievement [16], it reflects on how engaged students are well-adjusted academically. Adjustment is a key characteristic of PWB. Thus, it is likely that institutional support will enhance students’ PWB by positively influencing their engagement and adjustment levels. Therefore, we propose the following:

*Hypothesis 6: Academic engagement partially mediates the relationship between institutional support and psychological well-being of students in a team*.

Family and friends support are sources of social support that have strong influence on academic attitudes and behaviors of students [106]. They promote persistence in academic pursuits [107] and lower withdrawal from college studies [108]. Friendships with positive features such as intimacy and emotional support have positive effects on adjustment at school [109] reflecting on how well students have adapted to academic requirements and experience well-being.

FRS is positively associated with engagement levels of students [110]. This can be because of peer-related belongingness [111]. Family emotional support positively influences academic engagement levels [25]. Family support for learning in the form of academic and motivational support, goals and expectations, monitoring and supervision and learning resources is a significant factor underlying cognitive and psychological engagement of students [112].

The Broaden and Build theory [31] positive emotions and our responses to situations. It concludes that positive emotions help us adapt better to situations as they broaden one’s repertoire of responses to situations. This broadening of repertoire of responses in turn leads to building personal resources that influence study engagement positively [29]. Students who experience social support are also equipped with various psychological resources in the form of ‘psychological capital’, which as a whole is positively related to engagement [113].

Some of the critical outcomes of engagement are school completion [114] and persistence through college [115]. These reflect on students’ adaptation to academic requirements. Since adapting to the environment is key to psychological well- being, engagement and well-being can be positively related [116]. Thus, the positive emotions and psychological capital generated through FAS and FRS, are likely to enhance PWB through their influence on academic engagement and adaptation levels of students. Therefore, we propose the following:

*Hypothesis 7*: *Academic engagement partially mediates the relationship between family support and psychological well-being of students in a team*.
*Hypothesis 8*: *Academic engagement partially mediates the relationship between friends support and psychological well-being of students in a team*.

## Methodology

We collected data through a questionnaire survey designed in google forms after getting approval from the Institutional Review Board of the first author’s university (2020/10/01/EXP). We approached management students of Indian universities, informed them about our study objectives, and sent them the online survey after getting their verbal informed consent. The data was collected from them between December 2020 and April 2021. To prevent social desirability and acquiescence bias, no names were collected during the survey. The survey was completely anonymous and the authors had no way of identifying any individual through the survey during or after data collection. Students were asked to provide their responses based on team-based assignments in the most recent course they had attended. Team-based assignments are a critical component of the management education curriculum [17, 117]. The experience of working in team assignments is driven by the internal team environment, in terms of how the team interacts to complete the work. All responses had to be rated on a 7-point Likert scale ranging from 1 (*Strongly Disagree*) to 7 (*Strongly Agree*).

We collected 309 complete responses from students, comprising responses from 141 female and 168 male students. 142 students were from the undergraduate programs and 167 students were from the post-graduate programs. The average age of the respondents was 21.67 (s.d. = 2.92). 99 students had worked in faculty-assigned groups and 210 students had worked in self-created groups, in their most recent course.

We analyzed data through IBM SPSS 28 and IBM AMOS 28. Exploratory factor analysis (EFA) was used to remove items with factor loading less than .5.

### Measures

Measure for ITE was adapted from an established 10-item team environment scale [12]. The scale measured student’s perception of social support, shared purpose and ability to voice thoughts and opinions within the team. Sample item is “We discuss our team’s main tasks and objectives to ensure that we have a fair understanding”. EFA revealed one factor with acceptable factor loading for all items. The composite reliability for ITE is 0.91.

Measure for IS was adapted from a 5-item scale of perceived organizational support [118]. The scale measured the student’s perception of support provided by the educational institution. Sample item is “My institution cares about my opinions”.

Measure for FAS was adapted from a 5-item family support scale [119]. The scale measured the student’s perception of support provided by the family. Sample item is “My family gives me the energy to focus on my academics”.

Measure for FRS was adapted from a 6-item friends support scale [120]. The scale measured the student’s perception of support provided by the friends outside the team, and within or outside the educational institute. Sample item is “My friends understand what I am going through”.

For each of IS, FAS and FRS, EFA revealed one factor with acceptable factor loading for all items. We also conducted a combined EFA for the three variables, and found them to load on three different factors with no significant cross-loadings. The composite reliability for IS, FAS and FRS were 0.94, 0.92 and 0.92 respectively.

Measure for AE was adapted from a 7-item engagement scale [85]. The scale measured the student’s experience of engagement in the academic course. Sample item is “I usually look forward to most of my classes”. EFA revealed one factor with acceptable factor loading for all items. The composite reliability for AE is 0.87.

Measure for PWB was adapted from an 18-item well-being scale [121]. The scale measured the student’s experience of subjective well-being. Sample item is “I like most parts of my personality”. Items for all variables were retained post EFA, except two items of PWB that were deleted due to low factor loading. The composite reliability for PWB is 0.90. All the selected items had acceptable Cronbach Alpha reliability of more than 0.70 (Table 1)

**Table 1:** Reliability of variables.

S. No.	Variables	Α
A	Internal Team Environment	0. 91
B	Family Support	0.92
C	Friends Support	0.92
D	Institutional Support	0.94
E	Academic Engagement	0.87
F	Psychological Well Being	0.89

*N* = 309; Cronbach Alpha = α

Confirmatory factor analysis (CFA) was done using the selected items. Based on the results, we tested the hypotheses using path analysis. We used age, gender and formation process of groups (faculty-assigned or self-created) as control variables since they were expected to influence AE and PWB.

## Results

The means, standard deviation and correlation between variables are provided in Table 2. The results showed significant correlation between all the variables in the proposed model.

**Table 2:** Means, standard deviation and inter-correlations.

Variables	M	S.D.	1	2	3	4	5	6	7	8	9
Gender	0.46	0.50	1								
Age	21.67	2.92	-0.30**	1							
Group formation	0.32	0.47	-0.06	0.22**	1						
Team environment	5.65	0.87	0.15**	-0.07	-0.04	1					
Family support	5.34	1.38	0.01	0.10	0.01	0.25**	1				
Friends support	5.48	1.10	0.21**	-0.14*	-0.07	0.32**	0.38**	1			
Institutional support	4.14	1.48	0.03	-0.15**	-0.01	0.20**	0.32**	0.32**	1		
Academic engagement	4.98	1.03	-0.05	0.09	0.09	0.28**	0.37**	0.15**	0.39**	1	
Psychological well being	5.40	0.82	0.03	0.11	0.06	0.40**	0.51**	0.33**	0.29**	0.43**	1

*N* = 309. *p < 0.05

**p < 0.01

***p < 0.001. Two-tailed significance values are reported.

Since the data was based on self-reporting by respondents, we checked for common method variance using Harman’s single factor test in SPSS [122]. The unrotated factor solution using principal component analysis and involving 49 components revealed 9 distinct factors with the first factor explaining 25.9% of the variance in data. Thus, there was no evidence of common method bias [123].

Based on the selected items, we conducted CFA. The chi-square test for the measurement model was χ^2^ (1091) = 1957.57, p < .001. Also, it showed an acceptable fit with CMIN/df = 1.79, root-mean-square error of approximation (RMSEA) = 0.05, standardized root-mean-square residual (SRMR) = 0.06 and comparative fit index (CFI) = 0.91 [124].

We created alternate structural models to analyze different combinations of full mediation and partial mediation of AE for the independent variables (Table 3). The findings revealed that the proposed partial mediation model had the best fit, suggesting partial mediation of AE for all the independent variables.

**Table 3:** Alternate structural models.

Alternate Models	χ^2^ (df)	CMIN/df	Δχ^2^ (Δdf)	RMSEA	SRMR	CFI
**Proposed Model**	**1957.57 (1091)**	**1.794**		**.051**	**.062**	**.908**
Model 1	2005.99 (1094)	1.834	.040	.052	.077	.904
Model 2	1971.96 (1092)	1.806	.012	.051	.069	.907
Model 3	2031.57 (1095)	1.855	.049	.053	.092	.901

*N* = 309. Indices with minimal difference have been represented upto 3 places of decimal

Proposed model: Direct relation between TE & AE, FMS, FRS, ITS & AE, and AE & PWB + Direct relation between TE & PWB and FMS, FRS, ITS & PWB

Model 1: Direct relation between TE & AE, FMS, FRS, ITS & AE, and AE & PWB + Direct relation between TE & PWB

Model 2: Direct relation between TE & AE, FMS, FRS, ITS & AE, and AE & PWB + Direct relation between FMS, FRS, ITS & PWB

Model 3: Direct relation between TE & AE, FMS, FRS, ITS & AE, and AE & PWB

Df: Degrees of freedom; RMSEA: Root-mean-square error of approximation; SRMR: Standardized root-mean-square residual; CFI: Comparative fit index

TE: Team Environment; FMS: Family Support; FRS: Friends Support; ITS: Institutional Support; AE: Academic Engagement; PWB: Psychological Well-being

**Fig 2:**
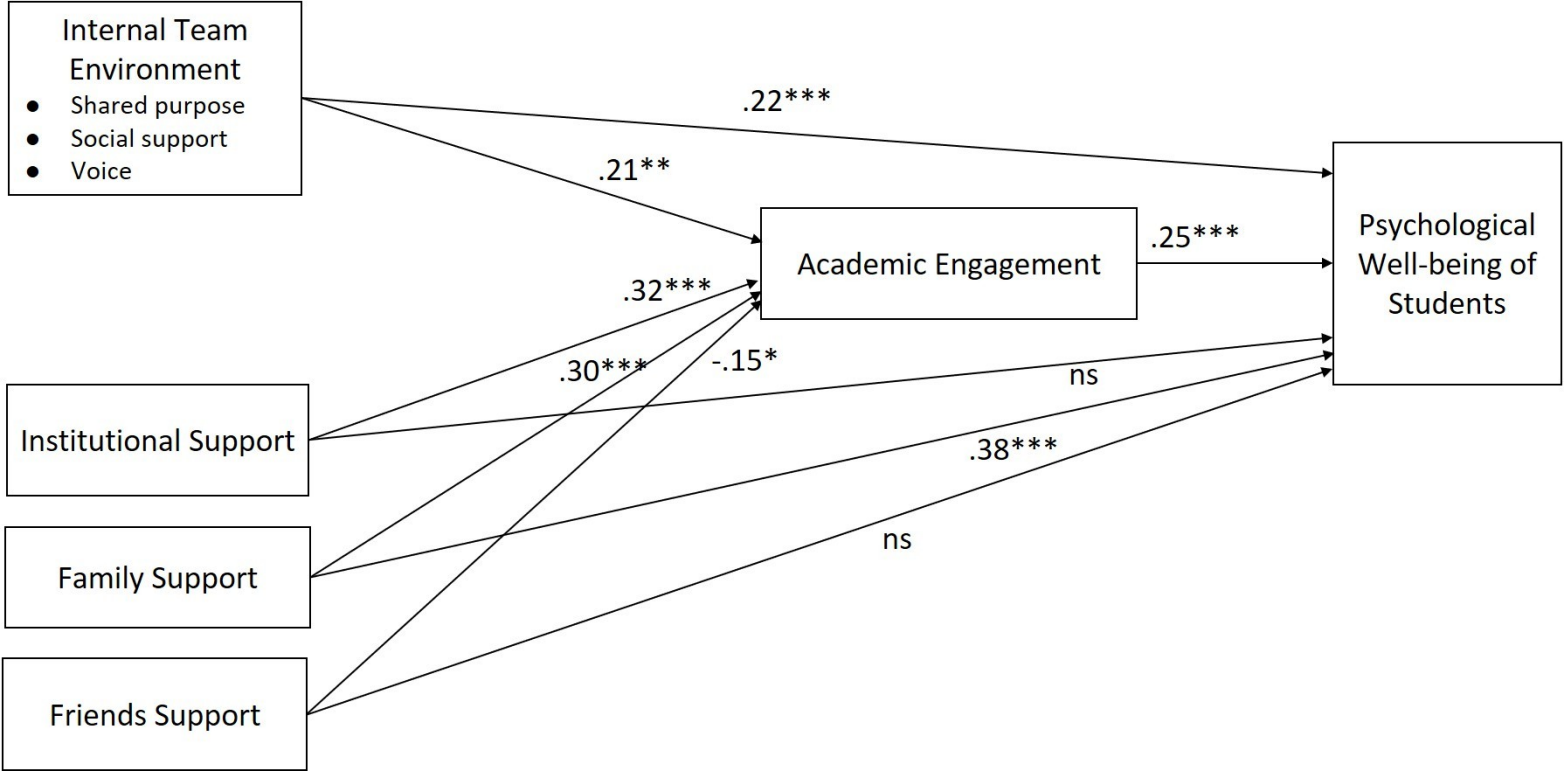
Path analysis results of the factors determining student psychological well-being.

We, therefore, proceeded to test all the hypotheses for the proposed model using hierarchical regression and path analysis. Results of path analysis are included in Fig 2.

Model 2 of hierarchical regression (Table 4) was used to test Hypotheses 1–4. Hypothesis 1 predicting that ITE is positively related to PWB (*β* = 0.27, *p <* .001) was supported. Hypothesis 2 predicting that IS is positively related to PWB (*β* = 0.11, *p <* .05) was supported. Hypothesis 3 predicting that FAS is positively related to PWB (*β* = 0.37, *p <* .001) was supported. However, FRS did not significantly relate to PWB (*β* = 0.09, *ns*) indicating lack of support for Hypothesis 4.

**Table 4:** Hierarchical regression for the proposed model.

Predictor Variables	Model 1	Model 2	Model 3
Control Variables			
Age	0.12*	0.11*	0.09
Gender	0.07	0.00	0.01
Group formation	0.03	0.05	0.03
Independent Variables			
Internal Team environment		0.27***	0.22***
Friends support		0.09	0.10*
Family support		0.37***	0.32***
Institutional support		0.11*	0.04
Mediating Variable			
Academic engagement			0.21***
R^2^	.02	.36***	.40***
ΔR^2^	.02	.35	.03
Adjusted R^2^	.01	.35	.38
F	1.7	24.68***	24.74***
ΔF	.29	12.6***	

*N* = 309. *p < 0.05

**p < 0.01

***p < 0.001.

All coefficients are standardized; One-tail significance values are reported.

Model 3 of hierarchical regression (Table 4) indicated that in the presence of AE as a mediating variable, ITE (*β* = 0.22, *p <* .001) and FAS (*β* = 0.32, *p <* .001) significantly related to PWB, but IS did not (*β* = 0.04, *ns*). Also, AE and PWB were positively related (*β* = 0.21, *p <* .001).

Path analysis presented similar results (Table 5). AE and PWB were positively related (*β* = 0.25, *p <* .001). ITE was positively related to AE (*β* = 0.21, *p <* .01) as well as PWB (*β* = 0.22, *p <* .001). Considering results of Hypothesis 1, this suggests partial mediation of AE for the relationship between ITE and PWB [125], thus supporting Hypothesis 5. IS was positively related to AE (*β* = 0.32, *p <* .001), but had insignificant relation with PWB (*β* = 0.04, *ns*). Considering results of Hypothesis 2, this suggests that AE fully mediates the relation between IS and PWB, thus partially supporting Hypothesis 6. FAS was positively related to AE (*β* = 0.30, *p <* .001), as well as PWB (*β* = 0.38, *p <* .001). Considering results of Hypothesis 3, this suggests that AE partially mediates the relation between FAS and PWB, thus supporting Hypothesis 7. The mediation of AE for the relationship between FRS and PWB (Hypothesis 8) was not supported since Hypothesis 4 was not supported. All the results of hypothesis testing are summarized in Table 6.

**Table 5:** Path analysis for the proposed model.

	Paths		*β*	*p*-value
TE	------>	AE	0.21	.001
FMS	<---	AE	0.30	***
FRS	<---	AE	-0.15	.027
ITS	<---	AE	0.32	***
AE	<---	PWB	0.25	***
TE	<---	PWB	0.22	***
FMS	<---	PWB	0.38	***
FRS	<---	PWB	0.05	.440
ITS	<---	PWB	0.04	.542

*N* = 309. ***p < 0.001; All coefficients are standardized.

TE: Team Environment; FMS: Family Support; FRS: Friends Support; ITS: Institutional Support; AE: Academic Engagement; PWB: Psychological Well-being

**Table 6:** Results of hypotheses testing.

Independent Variables	Internal Team Environment	Institutional Support	Family Support	Friends’ Support
Direct effect	*Positive (H1 supported)*	*Positive (H2 supported)*	*Positive (H3 supported)*	*Insignificant (H4 not supported)*
Mediation of Academic Engagement	*Partial mediation (H5 supported)*	*Full mediation (H6 partially supported)*	*Partial mediation (H7 supported)*	*H8 not supported*
Dependent Variable	Psychological Well-Being			

## Discussion

Student PWB is an important domain for study, not only from the standpoint of investigating its determinants, but also from the perspective of identifying mechanisms to improve it. Research in PWB of college students has only recently gained momentum in India. An Indian study on college students found a significant positive relationship between self-esteem and PWB [126]. Some other studies on college students in the Indian context found positive relationship of academic performance and task-oriented coping strategy with PWB [127], and negative relationship between excess internet use and PWB [128]. This paper indicates that ITE, IS and FAS are positively related to PWB of management students in India. Besides, AE partially mediates the relation of ITE and FAS with PWB, and fully mediates the relation of IS with PWB. The paper highlights the importance of team environment and support systems for the PWB of management students in higher education. It also suggests that the level of engagement students experience in their coursework can be closely related to their PWB. These findings extend the literature on PWB of students [93].

Our findings show that a positive ITE is positively related to students’ PWB, both directly, as well as through a positive relation with AE. Although working in teams is an integral part of the academic life of management students, very few studies have analyzed ITE in student teams. Besides, these studies have focused on understanding the phenomenon in the business domain [23], rather than the academic domain. Considering that collaboration and working in teams is a critical medium of student learning, the paper makes an important contribution to higher education literature, by exploring team environment in the academic domain and highlighting its significance for both academic engagement and well-being [129, 130] The findings thus also extend the empirical literature on ITE, which has focused on outcomes like shared leadership, effectiveness and performance [12, 23]. These results also add to the social interdependence theory and collaborative learning domain [21] by identifying the characteristics of the team context incorporated in ITE (shared purpose, social support, voice) that can lead to positive team experiences and higher well-being. Besides, these results contribute to the literature on self-determination theory by indicating the role that positive ITE may have, in influencing psychological well-being through satisfaction of relatedness and competence needs of the students.

Our results also show that IS is positively related to students’ PWB, and this relation is due to its positive relation with AE. This underscores the critical role of institutional support in the experience of students of higher education. Although there is literature indicating the effect of POS on PWB from the employee perspective [59], there are limited studies exploring this relation from the students’ perspective [131]. Besides, we do not know of any existing studies that have discussed the academic consequences of IS. By highlighting the importance of AE in determining the relation of IS and PWB of management students, this paper adds to the higher education literature [43, 91] as it contributes to Tinto’s interactionalist theory of individual student departure and Astin’s theory of student involvement, by showing the influence of institutional support on PWB through the mediating role of academic engagement.

Further, our results show that FAS is positively related to students’ PWB, and AE partially mediates the relationship. This underscores the importance of family support in academic engagement of management students, thus adding to the higher education literature [25, 91] and the family support literature in the education context [25]. Studies have shown that social support acts as a buffer in stressful situations [76] and enables personal adjustment [80]. It is possible that this happens because experience of support is likely to lead to generation of positive emotions. Previous research on Fredrickson’s broaden and build theory has shown that academic engagement of students experiencing positive emotions is enhanced through adaptive coping behaviors [132]. Therefore, our study contributes to the higher education literature on broaden and build theory by discussing the possible role of support provided by family in generating positive emotions, and in turn, the influence of these positive emotions on PWB through the mediating role of academic engagement. Studies on self-determination theory have discussed the role of parent support in satisfaction of basic psychological needs that in turn influence student’s academic motivation [133]. Our study contributes to this literature by showing the relationship of family support to students’ academic engagement.

Contrary to our hypothesis, our findings revealed that FRS is not related to students’ PWB Moreover, it is negatively related to AE (Table 5). A possible reason for these findings can be found when we look at past studies on the influence of classmate relationships on academic motivation and persistence, which have shown mixed results [133]. Friendships with classmates may not significantly influence academic engagement and performance when students have quality relationships with parents and teachers [134]. Another possible reason for lack of support for the hypotheses can be that different students in the sample considered different categories of friends, while responding to the questionnaire. Friends can be from the same course, batch, program, city, past school or neighbourhood. Different types of friends may impact engagement and well-being differently leading to unreliable findings. The results also suggest the possibility of negative influence of friends, in terms of distractions and misplaced goals, which can hamper students’ academic engagement. This opens up new avenues for research in the area of academic implications of support from different types of friends.

Our findings indicate the positive and mediating role of AE for the relation of formal and informal support systems like ITE, IS and FAS with PWB of management students. This implies that AE is desirable, not only for the performance of students, but also for well-being of students. Previous literature has found that AE leads to better academic progress [86], academic performance [87, 88] and commitment to academic activities [89]. However, limited studies have explored AE in the context of formal and informal support systems of management students [16], and their relation with PWB. Besides, no specific studies have explored the influence of ITE on engagement levels of students, although collaborative learning [52] and cooperative behaviors [16] have been found to act as antecedents for engagement levels among students. Our findings indicate the importance of team work in the direction of enhancing engagement levels of students, and the importance of engagement in enhancing the students’ PWB in higher education domain [16, 91]. The present study also contributes to the literature on ‘flow’, which is very similar in nature to ‘engagement’. Although flow has been studied in the higher education domain [135] there is no previous research discussing the influence of ITE, IS and FAS in the context of flow. The present study, while explaining the role of academic engagement in determining PWB due to support systems and ITE [100], also contributes to the literature on flow by exploring its possible antecedents.

### Practical implications

The findings of the paper can be utilized by the management of higher education institutions, faculty and students and their families. They indicate some actions that different stakeholders can undertake to promote both AE and PWB of students.

Educational institutions consider engagement and well-being of students as critical for their learning, growth and experience. They can facilitate these outcomes through certain initiatives. First, they can encourage faculty to design team-based activities and/or assignments. This would enable ample opportunities for students to work in teams. Students can also be encouraged to form their own groups, which is likely to promote positive ITE, at least initially. Second, institutions can create guidelines to promote positive ITE within student groups. For example, they can mandate the students to talk to their faculty, whenever they are facing challenges in the ITE. The faculty can help create norms for the teams, and encourage adherence to those norms. Peer feedback can help to maintain such norms. Third, institutions can strive towards a culture where students are more united, focused and goal-oriented, are willing and able to support each other when required, and feel psychologically safe and bold about sharing their ideas and concerns. Institutional leaders can play an important role in building such a culture by repeated communication of such values, and display of authenticity in their response to situations. Fourth, they can monitor the level and quality of support they provide to the students in terms of resources, course workload and schedule, subject matter expertise and administrative system and procedures. They can establish grievance handling cells to support the students when they experience problems related to infrastructure and amenities, for example, accommodation, food, classrooms, power, technology and library. Fifth, institutions can adopt academic advisory systems where nominated faculty mentors / advisors oversee the academic engagement and well-being of individual students assigned to them. The mentors / advisors can be responsible for suggesting techniques to students to improve their engagement. Institutions can also maintain a counselling department, which the mentors can recommend to the students, if they feel the need for it. Students should be able to have easy access to the qualified counsellors when they are facing mental health issues. All these policies can enhance student engagement and well-being.

The findings have implications for faculty too. Faculty can create positive team norms and actively engage with students, so as to ensure that members of each student team share a sense of purpose, provide psychological and emotional support to each other, and are comfortable and willing to use their voice whenever required.

Students can themselves drive outcomes beneficial for them. They can form teams (if they have the option), based on the criteria of a shared sense of purpose, and possibility of getting support and using voice within the team. If the students take the initiative to form teams, it is likely that they will choose peers whom they are comfortable with, leading to a higher probability of a positive internal team environment.

### Limitations and research avenues

We collected data from some universities that were accessible to us, which may raise questions about the representativeness of the results. Collecting data from diverse educational institutions situated in different cities and states, and having residential and non-residential campus, can make the sample more representative and the results more generalizable. It would also help to take care of any confounding effects due to the institutional and local culture, institutional leadership, policies and practices, course content and student profile. Moreover, such a study can be conducted across countries to identify if there is any influence of national culture.

Besides, we conducted a cross-sectional rather than a longitudinal study, which negates identification of causal relationships. Longitudinal research can help analyze the effect of formal and informal support received by a student in an educational institute at the beginning of a semester, on the academic engagement and psychological well-being in the middle and end of the semester.

In future, we can explore the hypothesized model in the context of non-management students across a wide range of disciplines, to identify variations if any. Besides, we can investigate differences in the model for management and non-management students. Further, we can examine whether different categories of friends (prior school, college, current institute etc.) affect students in the higher education domain differently, in order to verify the results of the current study.

In the current study, we have analyzed the internal team environment at the individual team member level as our primary interest was in analyzing the impact of the team member’s perception of the environment on the individual academic engagement and psychological well-being. Future studies can extend this study by considering a multi-level analysis that incorporates a team-level analysis of the internal team environment [136].

Future research can also consider additional determinants of students’ well-being in a team, like personality and diversity of team members, student related processes and policies, and institutional culture. Studies can also explore goal-orientation of students, and its impact on their engagement and well-being. Goal-orientation can be influenced by several factors including individual values, career goals, and even course work. Student perception and attitude towards the course work, faculty and institution can also be studied as part of the model, since they can affect engagement and well-being. Besides, tolerance of risk of academic failure can also be included in the model as risk tolerance impacts behavior [137].

Working in teams is an integral part of employees’ work life across industries. The current model can be tested in the context of organizations to understand the impact of teamwork and team dynamics on employee engagement, and their psychological well-being. This would be especially relevant for organizations that are engaged in team-oriented activities like total quality management [138]. The differential effect of organizational, peer and family support on employee engagement and well-being can also be examined. Considering that organizations are facing challenges in both engaging employees and managing their well-being, this research can provide some useful insights.

## Conclusion

This paper examines the relation between team and support related factors and academic engagement and consequently, psychological well-being of management students in India. Results based on survey data collected from students revealed that internal team environment, and institutional and family support are positively related to academic engagement and well-being, and academic engagement mediates the relation. However, friend support is negatively related to engagement and does not relate to well-being. Data for the current study was based on the sample of management students from a few universities in India. These findings contribute to theory by highlighting the relation amongst teamwork, support structures, academic engagement and well-being of management in higher educational institutions. Further research would be needed to examine the generalizability of the findings to non-management areas of the higher education domain and in other countries and cultures.

## Supporting information

S1 Dataset (XLS)
